# Extubation in the operating room results in fewer composite mechanical ventilation-related adverse outcomes in patients after liver transplantation: a retrospective cohort study

**DOI:** 10.1186/s12871-021-01508-1

**Published:** 2021-11-18

**Authors:** Yan Xu, Yiding Zuo, Li Zhou, Xuechao Hao, Xiao Xiao, Mao Ye, Lulong Bo, Chunling Jiang, Jiayin Yang

**Affiliations:** 1 Department of Anesthesiology, West China Hospital, Sichuan University & The Research Units of West China (2018RU012), Chinese Academy of Medical Sciences, Chengdu, 610041 China; 2 Department of Anesthesiology, Changhai Hospital, Naval Medical University, Shanghai, 200433 China; 3 Department of Liver Surgery and Liver Transplantation Center, West China Hospital, Sichuan University & The Research Units of West China (2018RU012), Chinese Academy of Medical Sciences, Chengdu, 610041 China

**Keywords:** liver transplantation, extubation in the operating room, adverse outcomes, propensity score matching

## Abstract

**Background:**

To investigate the effect of extubation in the operating room (OR) on mechanical ventilation-related adverse outcomes in patients who undergo liver transplantation.

**Methods:**

Patients who underwent liver transplantation between January 2016 and December 2019 were included. According to the timing of extubation, patients were divided into OR extubation group and intensive care unit (ICU) extubation group. The propensity score was used to match OR extubation group and ICU extubation group at a 1:2 ratio by demographical and clinical covariates. The primary outcome was a composite of mechanical ventilation-related adverse outcomes, including 30-day all-cause mortality, in-hospital acute kidney injury (stage 2 or 3), and in-hospital moderate to severe pulmonary complications. Secondary outcomes included in-hospital moderate to severe infectious complications, unplanned reintubation rates, ICU and postoperative hospital lengths of stay, and total hospital cost.

**Results:**

A total of 438 patients were enrolled. After propensity score matching, 94 patients were in OR extubation group and 148 patients were in ICU extubation group. Incidence of the composite mechanical ventilation-related adverse outcomes was significantly lower in OR extubation group than ICU extubation group, even after adjusting for confounding factors (19.1% *vs.* 31.8%; Odds Ratio, 0.509; 95% Confidence Index [CI], 0.274-0.946; *P*=0.031). The duration of ICU stay was much shorter in OR extubation group than ICU extubation group (median 4, Interquartile range [IQR] (3 ~ 6) *vs.* median 6, IQR (4 ~ 8); *P*<0.001). Meanwhile, extubation in the OR led to a significant reduction of total hospital cost compared with extubation in the ICU (median 3.9, IQR (3.5 ~ 4.6) 10000 US dollars *vs.* median 4.1, IQR (3.8 ~ 5.1) 10000 US dollars; *P*=0.021). However, there were no statistically significant differences in moderate to severe infectious complications, unplanned reintubation rates, and the length of postoperative hospital stay between groups.

**Conclusions:**

Among patients who underwent liver transplantation, extubation in the OR compared with extubation in the ICU, significantly reduced the primary composite outcome of 30-day all-cause mortality, in-hospital acute kidney injury (stage 2 or 3), or in-hospital moderate to severe pulmonary complications.

**Trial registration:**

The trial was registered at www.clinicaltrials.gov with registration number NCT04261816. Retrospectively registered on 1st February 2020.

**Supplementary Information:**

The online version contains supplementary material available at 10.1186/s12871-021-01508-1.

## Introduction

Liver transplantation has been established as the most effective treatment for acute and chronic end-stage liver diseases [1]. Delaying extubation to maintain postoperative ventilatory support is the routine strategy for these patients [2–4]. Previous studies have suggested that postoperative mechanical ventilation with sedation may decrease surgical stress response and may improve hemodynamic stability, thereby facilitating early recovery in patients after liver transplantation [5]. Nonetheless, mechanical ventilation can provide a direct passageway for pathogens into the lungs, and reduce the mucosal immune barrier function, which may subsequently increase the risk of postoperative pulmonary complications [6, 7]. Moreover, increasing evidence showed that mechanical ventilation, through reducing cardiac output and increasing central venous pressure and diminishing renal blood flow [8], is associated with a threefold increase in the risk of acute kidney injury (AKI) [9]. Additionally, postoperative mechanical ventilation also increases medical care cost [10, 11] and postoperative mortality [12, 13].

Over the years, many studies have reported on early extubation after liver transplantation, mainly about investigating the association between extubation in the OR and postoperative pulmonary complications [14–16]. Yet, the relationship between extubation in the OR and other adverse outcomes, *e.g.*, 30-day all-cause mortality and in-hospital AKI, which is strongly associated with mechanical ventilation [9], remains undefined. Thus, we performed this retrospective cohort study with propensity score matching to investigate the effect of extubation in the OR after liver transplantation on a composite of mechanical ventilation-related adverse outcomes, which consists of 30-day all-cause mortality, in-hospital AKI (stage 2 or 3), and in-hospital moderate to severe pulmonary complications.

## Methods

### Ethical considerations

The medical ethics committee of the Institutional Review Board of West China Hospital, Sichuan University, reviewed and approved this retrospective data-only study with a waiver of informed consent (No. 2020–014), and it was performed in accordance with Strengthening the Reporting of Observational Studies in Epidemiology (STROBE) [17] criteria (see Additional file 1: Table S1)

### Patients

This retrospective cohort study involved patients from West China Hospital, Sichuan University. The study period was from January 1, 2016, to December 31, 2019, with the following inclusion criteria: (1) age ≥18 years; (2) those who underwent liver transplantation. Patients were excluded if they met any of the following criteria: (1) re-transplantation; (2) multi-visceral transplantation; (3) intraoperative death; (4) severe encephalopathy (West Haven criteria III or IV); (5) those who were already intubated before liver transplantation; (6) incomplete clinical data.

Patients were divided into two groups based on the timing of their extubation: those who were extubated in the OR were grouped into the OR extubation group, and those who were extubated in the ICU were grouped into the ICU extubation group.

### Anesthesia Procedure

All patients received an orthotopic liver transplantation (piggy-back or classic) procedure under general anesthesia. Surgery and anesthesia were consistently provided by transplant surgeons and anesthesiologists with at least 5 years’ experience in liver transplantation, respectively.

In all cases, balanced anesthesia with inhalational agents, opioids, and muscle relaxants were performed under routine monitoring. A standardized ventilation setting protocol of tidal volume (6 to 8 mL/kg) and respiratory rates (10 to 16 breaths/min) were given to maintain end-tidal carbon dioxide pressures (ETCO_2_) of 35 to 45 mm Hg. Intraoperative fluid, blood product transfusion, and vasopressors were managed based on the anesthesiologist's clinical judgment utilizing intraoperative thermoelectrometry, arterial blood gases, and coagulation test results.

The decision to extubate the trachea was made by the individual attending anesthesiologist in consultation with surgeons towards the end of surgery. The criteria for extubation were following: patient being awake, obedience to verbal commands, current volume above 6 mL/kg, breath rate between 10 to 18 per minute, E_T_CO_2_ between 35 to 45 mm Hg, and O_2_ saturation above 95% (with FiO_2_ ≤ 40 %), hemodynamic stability, normothermia, a complete reversal of neuromuscular blockade and adequate hemostasis in the surgical field. After extubation, the patient was transferred to the ICU for further care.

Patients without plans for extubation in the OR or who failed to fulfill the criteria for OR extubation were transferred to the ICU for mechanical ventilation support. In the ICU, weaning from mechanical ventilation was left to the discretion of the ICU attending physician.

### Definitions of outcomes

The primary outcome was a composite of mechanical ventilation-related adverse outcomes, which consisted of any of the following: 30-day all-cause mortality, in-hospital AKI, or in-hospital moderate to severe pulmonary complications. AKI (stage 2 or 3) was defined according to Kidneys Disease improving Global Outcomes criteria [18] (see Additional file 2:Table S2). The pulmonary complications were defined as the first occurrence of pulmonary infection, respiratory failure, and pleural effusion if these occurred between ICU admission and hospital discharge. The pulmonary complication was defined and graded as mild, moderate, or severe, as described by the European Perioperative Clinical Outcomes definitions [19] (see Additional file 3:Table S3).

The secondary outcomes included in-hospital moderate to severe infectious complications, unplanned reintubation rates, ICU and postoperative hospital lengths of stay, and total hospital cost. The infectious complications included any of the followings: new-onset surgical site infection (superficial/deep, organ/space), pulmonary infection, or bloodstream infection. The infectious complications were defined and graded as mild, moderate, or severe, as described by the European Perioperative Clinical Outcomes definitions [19] (see Additional file 3:Table S3). The total hospital cost included preoperative imaging and laboratory examination, drugs and intraoperative surgery-related and anesthesia-related, and treatment in the ICU and surgical complications after surgery.

### Data Collection

Specially trained research personnel collected all data from the electronic health record system. Preoperative data included age, sex, body-mass-index (BMI), smoking history, drinking history, American Society of Anesthesiologists (ASA) classification, comorbidities (including cardiovascular disease, respiratory disease, stroke, renal dysfunction, and diabetes), etiology of end-stage liver diseases, model for end-stage liver disease (MELD) score, preoperative artificial liver support and basic oxygen saturation (SpO_2_). The MELD score was calculated using the following formula: *[0.957 x log*_*e*_ *(creatinine mg/dl) + 0.378 x log*_*e*_ *(bilirubin mg/dl) + 1.120 x log*_*e*_ *(INR) +0.643] x 10*. In addition, preoperative laboratory data, such as hemoglobin, white blood cell (WBC), platelet count, prothrombin time (PT), activated partial thromboplastin time (APTT), international normalized ratio (INR), alanine transaminase, total bilirubin, and albumin were also collected. The intraoperative findings included duration of surgery, urine output, the volume of packed red blood cells (PRBC) transfused, the maximal dose of vasopressors, and the maximal lactate level. The postoperative variables included the postoperative complications that occurred between ICU admission and hospital discharge, tracheal extubation time, unplanned reintubation rates, length of ICU and postoperative hospital stay, total hospital cost, and death at 30 days. The complications were examined by 2 anesthesiologists, documented in the electronic health record system and adjudicated by anesthesiologists trained in outcome definitions and unaware of the patients’ groupings.

### Data analysis

Categorical variables, which were presented in percentages, were analyzed using the χ^2^ test or Fisher’s exact test. Continuous variables with normal distribution were reported as mean ± standard deviation (SD) and non-normally distributed variables as the median and interquartile range (IQR), were compared using independent Student’s t-test or Mann–Whitney U test.

We used propensity score matching to reduce potential bias and confounding factors. We took all reported factors that were considered clinically important as covariates in the model to further reduce potential confounding. Final covariates included age, sex, BMI, smoking history, drinking history, ASA classification, comorbidities (including cardiovascular disease, respiratory disease, stroke, renal dysfunction, and diabetes), etiology of end-stage liver diseases, MELD score, preoperative artificial liver support, basic SpO_2_, preoperative laboratory data (including hemoglobin, WBC, platelet count, PT, APTT, INR, alanine transaminase, total bilirubin, and albumin) and intraoperative findings (including duration of surgery, urine output, the volume of PRBC transfused, the maximal dose of vasopressors and the maximal lactate level). A logistic regression model was used to calculate propensity scores predicting the probability of receiving OR extubation. Matching was performed with the use of a 1:2 matching protocol without replacement (greedy nearest neighbor method), with a caliper width equal to 0.2 of the standard deviation of the logit of the propensity score [20]. We used the standardized difference to assess balance at baseline in both groups [21]. The standardized differences of less than 10% for a given covariate indicated a relatively small imbalance [21].

Association between extubation in the OR and the occurrence of composite mechanical ventilation-related adverse outcomes was assessed by logistic regression analysis. Factors with P<0.10 in univariable analyses or those considered clinically important were included in a multivariable logistic regression model to determine the risk-adjusted association between OR extubation and the risk of composite adverse outcomes development using a backward procedure. The multivariable logistic regression analysis was used to evaluate the unmatched data and matched data. In addition, we also assessed the association between baseline variables and the selection of OR extubation in all patients with logistic regression models. Factors that were considered clinically important or significant (*P* < 0.1) in univariate analysis were included in a multivariable logistic regression model to determine the independent factors that affected OR extubation by using a Enter procedure.

All reported *P*-values were two-sided, and a *P*-value <0.05 was considered statistically significant. Data were analyzed using SPSS® Statistics for Windows® version 22.0 (IBM).

## Results

### Patient Population

**Fig. 1:**
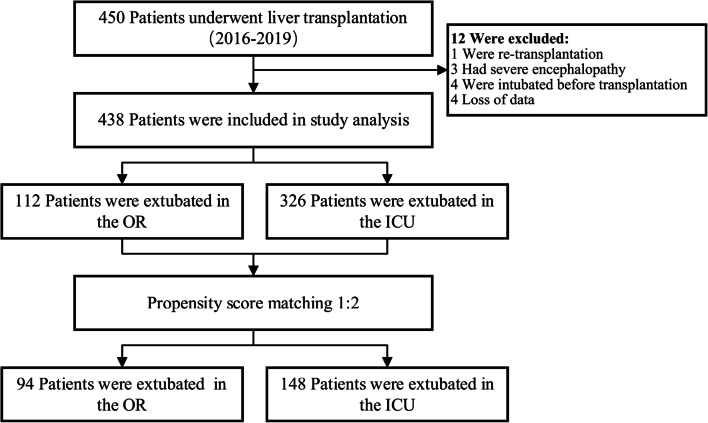
Study Flow-Chart. (OR, operating room; ICU, intensive care unit)

Among 450 patients who were assessed for eligibility between January 2016 and December 2019; 12 patients were excluded from the study for the following reasons: re-transplantation (1 case), severe encephalopathy (3 cases), already intubated before liver transplantation (4 cases), and loss of data (4 cases) (Fig. 1). Consequently, 438 patients were included in the study (Fig. 1). After propensity score matching, 94 patients extubated in the OR were matched to 148 patients extubated in the ICU.

### Extubation Status

In the entire cohort, 112 (25.6%) patients were extubated in the OR, 326 (74.4%) patients received ICU extubation. The median time of extubation was 12h following surgery (Table 1).

**Table 1:** Patient selections

Group	Before matching			After matching		
	n	Percent	Median time and range of extubation	n	Percent	Median time and range of extubation
**OR extubation group**	112	25.6	/	94	38.8	/
**ICU extubation group**	326	74.4	12 (8 ~ 19)	148	61.2	11 (8 ~ 18) h
Extubation in ICU (within 24h)	273	83.7	11 (8 ~14)	133	89.9	11 (7-14) h
Extubation in ICU (> 24h)	53	16.3	39 (32 ~ 54)	15	10.1	33 (30-48) h

Data are presented as median (interquartile range), or a number of patients (percentage), respectively

*OR* Operating room, *ICU* Intensive care unit

In the matched cohort, 94 (38.8%) patients received OR extubation, 148 (61.2%) patients were extubated in the ICU. The median time of extubation was 11h following surgery (Table 1)

### Baseline Characteristics

The baseline characteristics are presented in Table 2. Before propensity-score matching, the mean age of patients was 49 years (SD, 10) in the OR extubation group, and 51 years (SD, 10) in the ICU extubation group (*P*=0.169). Males made up 84.8% of the OR extubation group and 74.8% of the ICU extubation group (*P*=0.029). There were differences in some baseline variables between the two groups. After propensity score matching, almost all baseline variables were well balanced amongst the groups (Table 2). The standardized differences were < 10.0% for most variables. Patients in the OR extubation group were younger (49±10 *vs.* 51±10, Standardized Difference 11.8%) and had slightly higher preoperative albumin levels (38±6 *vs.* 37±7, Standardized Difference 10.8%) than the ICU extubation group. The estimated propensity scores before and after matching are shown in Histograms (see Additional file 4: Fig. S1).

**Table 2:** Baseline Characteristics Before and After Propensity Score Matching

Baseline	Original Cohort		***P***-Value	|Standardized Difference(%)|	Propensity-matched Cohort		***P***-Value	|Standardized Difference(%)|
	OR Extubation	ICU Extubation			OR Extubation	ICU Extubation		
	***n***=112	***n***=326			***n***=94	***n***=148		
**Age (y)**	49±10	51±10	0.169	15.1	49±10	51±10	0.260	11.8
**Sex**			0.029				0.680	
Male	95 (84.8%)	244 (74.8%)		27.7	80 (85.1%)	123 (83.1%)		1.5
Female	17 (15.2%)	82 (25.2%)		27.7	14 (14.9%)	25 (16.9%)		1.5
**BMI (kg/m**^**2**^**)**	22.9±2.8	22.7±3.4	0.440	8.9	22.9±2.8	22.8±3.5	0.755	2.5
**Smoking**	39 (34.8%)	136 (41.7%)	0.199	14.4	36 (38.3%)	56 (37.8%)	0.943	1.1
**Drinking**	26 (23.2%)	90 (27.6%)	0.363	10.4	24 (25.5%)	30 (20.3%)	0.338	10.0
**ASA-classification**			<0.001				0.051	
ASA *II*	5 (4.5%)	12 (3.7%)		53.3	2 (2.1%)	10 (6.8%)		1.1
ASA III	82 (73.2%)	160 (49.1%)		54.3	68 (72.3%)	86 (58.1%)		0.0
ASA IV	25 (22.3%)	153 (46.9%)		58.8	24 (25.5%)	52 (35.1%)		4.0
ASA V	0	1 (0.2%)		/	0	0	/	/
**Comorbidity**								
Cardiovascular disease^a^	0	5 (1.5%)	0.422	/	0	0	/	/
Respiratory disease^b^	3 (2.7%)	14 (4.3%)	0.631	10.0	3 (3.2%)	4 (2.7%)	>0.999	3.0
Stroke	0	0	/	/	/	/	/	/
Renal dysfunction (eGFR < 60 ml/min/1.73m^2^)	3 (2.7%)	23 (7.1%)	0.091	2.7	3 (3.2%)	5 (3.4%)	>0.999	3.3
Diabetes	10 (8.9%)	34 (10.4%)	0.648	5.2	8 (8.5%)	17 (11.5%)	0.458	5.6
**Etiology of end-stage liver disease**			0.246				0.947	
Cirrhosis	78 (69.6%)	206 (63.2%)		14.0	63 (67.0%)	97 (65.5%)		1.2
Liver cancer	25 (22.3%)	75 (23.0%)		1.6	23 (24.5%)	39 (26.4%)		4.0
Other	9 (8.0%)	45 (13.8%)		21.0	8 (8.5%)	12 (8.1%)		4.0
**MELD score**	13 (9 ~ 18)	16 (10 ~ 24)	0.003	35.5	13 (9 ~ 19)	13 (9 ~ 20)	0.713	1.8
**Preoperative artificial liver support**	5 (4.5%)	31 (9.5%)	0.094	24.3	5 (5.3%)	8 (5.4%)	0.977	0.0
**Basic SpO**_**2**_	100 (98 ~ 100)	99 (98 ~ 100)	0.079	9.4	99 (98 ~ 100)	98 (97 ~ 100)	0.119	1.5
**Preoperative laboratory data**								
Hemoglobin (g/L)	118±29	110±27	0.015	25.5	118±29	118±27	0.940	0.1
WBC (× 10^9^/L)	4.0 (2.9 ~ 5.6)	4.4 (3.1 ~ 6.5)	0.212	18.0	3.9 (3.0 ~ 5.6)	4.2 (3.0 ~ 6.1)	0.737	0.9
Platelet count (× 10^9^/L)	72 (41 ~ 115)	67 (43 ~ 106)	0.598	2.5	73 (43 ~ 120)	66 (43 ~ 104)	0.482	6.7
PT (s)	14.4 (12.8 ~ 17.2)	15.5 (13.4 ~ 20.3)	0.001	63.5	14.4 (12.8 ~ 17.6)	14.6 (13.0 ~ 17.4)	0.600	3.6
APTT (s)	34.4 (30.5 ~ 40.3)	37.0 (31.4 ~ 49.5)	0.013	36.4	35.3 (31.0 ~ 41.7)	34.5 (29.4 ~ 43.5)	0.691	4.3
INR	1.23 (1.10 ~ 1.49)	1.34 (1.16 ~ 1.83)	0.001	56.2	1.23 (1.10 ~ 1.55)	1.28 (1.14 ~ 1.53)	0.497	3.4
Alanine transaminase (IU/L)	34.5 (24.0 ~ 56.8)	39.0 (25.0 ~ 62.2)	0.124	22.5	34.5 (24.0 ~ 59.0)	39.0 (25.0 ~ 61.8)	0.381	2.6
Total bilirubin (μmol/L)	33.6 (14.5 ~ 145.8)	60.8 (20.6 ~ 223.4)	0.009	20.6	34.5 (14.3 ~ 155)	33.6 (17.8 ~ 156.2)	0.725	2.9
Albumin (g/L)	38±6	36±7	<0.001	42.6	38±6	37±7	0.299	10.4
**Intraoperative findings**								
Duration of surgery (h)	6.4 (6.0 ~ 7.0)	7.0 (6.0 ~ 9.0)	<0.001	61.6	7.0 (6.0 ~ 7.3)	7.0 (6.0 ~ 8.0)	0.629	3.8
Urine output (mL)	1017 (800 ~ 1500)	900 (500 ~ 1400)	0.026	21.3	1000 (800 ~ 1450)	1000 (600 ~ 1622)	0.643	1.6
Packed red blood cell transfusion (U)	4 (0 ~ 8)	9 (4 ~ 14)	<0.001	>100	5 (0 ~ 9)	6 (3 ~ 9)	0.155	5.9
Maximal dose of vasopressors^c^ (μg/kg/min)	0.08 (0.05 ~ 0.15)	0.10 (0.07 ~ 0.20)	<0.001	45.2	0.10 (0.05 ~ 0.15)	0.10 (0.05 ~ 0.15)	0.298	7.0
Maximal lactate level (mmol/L)	5.6 (4.4 ~ 8.6)	7.5 (5.0 ~ 11.2)	<0.001	46.6	6.4 (4.4 ~ 9.4)	6.4 (4.3 ~ 9.2)	0.930	1.0

Data are presented as mean ± SD, median (interquartile range), or a number of patients (percentage) and compared by independent samples t-test, Mann–Whitney U test or χ^2^ test/Fisher’s exact test, respectively

*OR* Operating room, *ICU* Intensive care unit, *BMI* Body mass index, *ASA* American Society of Anesthesiologists, *MELD* Model for End-Stage Liver Disease, *SpO*_*2*_ Oxygen saturation, *WBC* White blood cell, *PT* Prothrombin time, *APTT* Activated partial thromboplastin time, *INR* International normalized ratio

^a^ Including hypertension, coronary artery disease, congenital heart disease

^b^ Including chronic obstructive pulmonary disease, pulmonary infection, pleural effusion

^c^ Including epinephrine, norepinephrine

### Primary Outcome

The composite mechanical ventilation-related adverse outcomes were identified in 26.8% (65/242) of the matched patients. Noteworthily, patients who were extubated in the OR had a lower incidence of the composite mechanical ventilation-related adverse outcomes (19.1%) compared to patients extubated in the ICU (31.8%), and were associated with a significantly decreased risk of composite mechanical ventilation-related adverse outcomes (Odds Ratio, 0.509; 95% CI, 0.274 ~ 0.946; *P*=0.031, Table 3). Unfortunately, when analyzing each individual event that made up the composite outcome, no significant differences were found between OR extubation and ICU extubation groups in AKI (stage 2 or 3) (*P*=0.181), pulmonary complications (*P*=0.306), and 30-day all-cause mortality (*P*=0.081), as shown in Table S4 (see Additional file 5).

**Table 3:** Outcomes Before and After Propensity Score Matching

Outcome	Original Cohort		Odds Ratio (95%CI)	***P***-Value	Propensity-matched Cohort		Odds Ratio (95%CI)	***P-***Value
	OR Extubation	ICU Extubation			OR Extubation	ICU Extubation		
	***n***=112	***n***=326			***n***=94	***n***=148		
**Primary outcome**								
30-day all-cause mortality, AKI (stage II or III) and Moderate-severe pulmonary complications	19 (17.0%)	125 (38.3%)	0.329 (0.191 ~ 0.565)	<0.001	18 (19.1%)	47 (31.8%)	0.509 (0.274 ~ 0.946)	0.031
**Secondary outcomes**								
Moderate to severe infectious complications	9 (8.0%)	65 (19.9%)	0.351 (0.169 ~ 0.731)	0.004	9 (9.6%)	23 (15.5%)	0.575 (0.254 ~ 1.304)	0.182
ICU length of stay	4 (3 ~ 5)	6 (4 ~ 10)	/	<0.001	4 (3 ~ 6)	6 (4 ~ 8)	/	<0.001
Postoperative hospital length of stay	12 (9 ~ 17)	15 (11 ~ 22)	/	0.001	12 (10 ~ 17)	13 (10 ~ 21)	/	0.063
Unplanned reintubation	7 (6.3%)	53 (16.3%)	0.343 (0.151 ~ 0.779)	0.008	6 (6.4%)	20 (13.5%)	0.436 (0.168 ~ 1.130)	0.081
Total hospital cost (10,000 US dollars)	3.9 (3.5 ~ 4.5)	4.4 (3.9 ~ 5.4)	/	<0.001	3.9 (3.5 ~4.6)	4.1 (3.8 ~ 5.1)	/	0.021

Data are presented as mean ± SD, median (interquartile range), or a number of patients (percentage) and compared by independent samples t-test, Mann-Whitney U test, or chi-square test/Fisher’s exact test, respectively

*OR* Operating room, *ICU* Intensive care unit, *CI* Confidence Index, *AKI* Acute kidney injury

### Secondary outcomes

After matching, the ICU length of stay decreased from the median (1^st^ quartile to 3^rd^ quartile) 6 (4 to 8) days in the ICU extubation group to 4 (3 to 6) days in OR extubation group (*P*<0.001) (Table 3). Moreover, extubation in the OR led to a significant reduction of total hospital cost compared with extubation in the ICU (median 3.9, IQR (3.5 ~ 4.6) 10000 US dollars *vs.* median 4.1, IQR (3.8 ~ 5.1) 10000 US dollars; *P*=0.021). In addition, there were no significant differences in moderate to severe infectious complications (9.6% *vs* 15.5%; Odds Ratio, 0.575; 95% CI, 0.254~ 1.304; *P*=0.182), postoperative hospital length of stay (median 12, IQR (10 ~ 17) *vs* median 13, IQR (10 ~ 21); *P*=0.063) between OR extubation group and ICU extubation group (Table 3).

The incidence of reintubation in OR extubation group was not increased when compared to that in the ICU group (*P*=0.081) (Table 3). A total of 26 patients (10.7%) required tracheal reintubation during hospitalization. The causes of reintubation in the OR extubation group were respiratory distress (*n* = 1 patient), reoperation (*n* = 4 patients) and cardiovascular instability (*n* = 1 patient) (see Additional file 6: Table S5); 20 patients in the ICU extubation group were reintubated due to altered mental status (*n* = 1 patient), respiratory distress (*n* = 5 patients), reoperation (*n* = 11 patients) and cardiovascular instability (*n* = 3 patients) (see Additional file 6: Table S5).

### Association between extubation in the OR and the composite mechanical ventilation-related adverse outcomes

To preserve the stability of the multivariable model, 9 variables that were identified by univariable analysis (*P*<0.10) or considered clinically important were included in a multivariable logistic regression model. After correction with confounding factors, extubation in the OR was associated with a significantly decreased risk of the composite mechanical ventilation-related adverse outcomes (Odds Ratio, 0.518; 95%CI, 0.290 ~ 0.924; *P*=0.026) (Table 4). In matched patients, after correction with confounding factors, extubation in the OR remained associated with a significant reduction of the composite mechanical ventilation-related adverse outcomes (Odds Ratio, 0.514; 95%CI, 0.270 ~ 0.981; *P*=0.044) (Table 4).

**Table 4:** Association between extubation in the OR and composite mechanical ventilation-related adverse outcomes^a^

Characteristics	All recipients Univariable ***P*** valve	Multivariable analysis Odds Ratio (95%CI)	***P*** valve	Matched recipients Univariable ***P*** valve	Multivariable analysis Odds Ratio (95%CI)	***P*** valve
**Sex**	0.029	—	—	0.68	—	—
**ASA-classification (n), %**	<0.001	0.488 (0.163~1.461)	0.200	0.051	0.640 (0.163~2.513)	0.523
**Renal dysfunction (eGFR <60ml/min/1.73m**^**2**^**)**	0.091	2.161 (0.927~5.037)	0.074	>0.999	0.680 (0.139~3.316)	0.633
**MELD score**	0.003	0.999 (0.966~1.033)	0.963	0.713	1.022 (0.987~1.058)	0.228
**Preoperative artificial liver support**	0.094	1.129 (0.515~2.474)	0.763	0.977	3.645 (0.680~19.535)	0.131
**Basic SpO2**	0.079	0.858 (0.769~0.957)	0.006	0.119	0.844 (0.720~0.990)	0.037
**Preoperative-Hemoglobin (g/L)**	0.015	—	—	0.940	—	—
**PT (s)**	0.001	—	—	0.600	—	—
**APTT (s)**	0.013	—	—	0.691	—	—
**INR**	0.001	—	—	0.497	—	—
**Total bilirubin (μmol/L)**	0.009	—	—	0.725	—	—
**Albumin (g/L)**	<0.001	—	—	0.299	—	—
**Duration of surgery (h)**	<0.001	1.091 (0.963~1.235)	0.171	0.629	1.054 (0.857~1.296)	0.617
**Urine output**	0.026	—	—	0.643	—	—
**Packed red blood cell transfusion (U)**	<0.001	1.083 (1.052~1.115)	<0.001	0.155	1.085 (1.025~1.148)	0.005
**Maximal vasopressors need (μg/kg/min)**	<0.001	—	—	0.298	—	—
**Maximal lactate (mmol/L)**	<0.001	1.041 (0.992~1.092	0.102	0.930	1.069 (0.996~1.146)	0.064
**Extubation**	—	0.518 (0.290~0.924)	0.026	—	0.514 (0.270~0.981)	0.044

*ASA* American Society of Anesthesiologists, *MELD* Model for End-Stage Liver Disease, *SpO2* Oxygen saturation, *PT* Prothrombin time, *APTT* Activated partial thromboplastin time, *INR* International normalized ratio, *CI* Confidence Index

^a^Composite mechanical ventilation-related adverse outcomes includes 30-day all-cause mortality, in-hospital acute kidney injury (stage 2 or 3), or in-hospital moderate to severe pulmonary complications.

### Factors associated with OR extubation

In the multivariate logistic regression analysis, 4 parameters were selected as independent predictors of OR extubation after liver transplantation (Table 5): smoking history (Odds Ratio, 0.602; 95%CI, 0.363 ~ 0.996;*P*=0.048), duration of surgery (Odds Ratio, 0.772; 95%CI, 0.655-0.911; *P*=0.002), perioperative PRBC transfusion units (Odds Ratio, 0.886; 95%CI, 0.844-0.929; *P*<0.001) and perioperative maximal dose of vasopressors (Odds Ratio, 0.050; 95% CI, 0.004-0.616; *P*=0.019). Patients with smoking history were less likely to undergo OR extubation than those without smoking history. A 1 hour increase in the duration of surgery decreased the chance for OR extubation by 22.8%, transfusion of 1 unit of PRBC decreased the chance for OR extubation by 11.4%, and 1μg/kg/min increase in the vasopressors dose decreased the chance for OR extubation by 95%.

**Table 5:** Multivariate logistic regression analysis to evaluate independent factors for OR extubation

Variable	Univariable analysis			Multivariate logistic analysis		
	Odds Ratio	95%CI	***P*** valve	Odds Ratio	95%CI	***P*** valve
**Age (y)**	0.986	0.965-1.006	0.169	0.987	0.963-1.011	0.279
**Sex**						
Female	Ref			Ref		
Male	1.878	1.058-3.333	0.031	1.708	0.819-3.562	0.153
**BMI (kg/m**^**2**^**)**	1.024	0.959-1.093	0.48	1.021	0.942-1.106	0.614
**Smoking**						
No	Ref			Ref		
Yes	0.746	0.477-1.167	0.199	0.602	0.363-0.996	0.048
**ASA-classification**						
ASA II	Ref					
ASA III	1.230	0.419-3.610	0.706	/	/	>0.999
ASA IV	0.392	0.127-1.209	0.103	/	/	>0.999
ASA V	0.000	0	>0.999	/	/	>0.999
**Cardiovascular disease**						
No	Ref					
Yes	0.000	/	>0.999	/	/	>0.999
**Respiratory disease**						
No	Ref			Ref		
Yes	0.613	0.173-2.175	0.449	0.838	0.204-3.444	0.806
**MELD score**	0.963	0.939-0.988	0.004	1.017	0.974-1.061	0.443
**Preoperative artificial liver support**						
No	Ref			Ref		
Yes	0.445	0.169-1.173	0.102	0.502	0.129-1.953	0.320
**Preoperative-Hemoglobin (g/L)**	1.010	1.002-1.018	0.015	0.991	0.980-.002	0.119
**Preoperative-WBC (× 10**^**9**^**/L)**	0.946	0.875-1.023	0.162	0.942	0.850-1.043	0.250
**Preoperative-Albumin (g/L)**	1.060	1.025-1.095	0.001	1.022	0.975-1.070	0.365
**Duration of surgery (h)**	0.739	0.641-0.852	<0.001	0.772	0.655-0.911	0.002
**Urine output (mL)**	1.000	1.0000-1.0001	0.042	1.000	1.000-1.001	0.028
**Packed red blood cell transfusion (U)**	0.867	0.829-0.906	<0.001	0.886	0.844-0.929	<0.001
**Maximal vasopressors need (μg/kg/min)**	0.017	0.001-0.197	0.001	0.050	0.004-0.616	0.019

*CI* Confidence Index, *OR* Operating room, *BMI* Body mass index, *ASA* American Society of Anesthesiologists, *MELD* Model for End-Stage Liver Disease, *WBC* White blood cell

## Discussion

Our analysis demonstrates that patients extubated in the OR were associated with a lower incidence of composite mechanical ventilation-related adverse outcomes, shorter ICU length of stay, and lower total hospital cost compared with ICU extubation. Unfortunately, extubation in the OR did not lead to a superior effect in moderate to severe infectious complications and postoperative hospital length of stay.

Previous studies have shown that postoperative mechanical ventilation, as the routine management strategy for most patients undergoing liver transplantation, can significantly increase the incidence of pulmonary complications [14–16]. Furthermore, mechanical ventilation has also been associated with a threefold increase in the risk of AKI [9]. Yet, it remains unclear whether these adverse outcomes can be reduced by shortening the time of mechanical ventilation through extubation in the OR. Consequently, we performed this retrospective study to explore whether extubation in the OR was associated with a lower incidence of aforementioned adverse outcomes. Considering some baseline variables that might influence the development of adverse outcomes were different between patients extubated in the OR and those extubated in the ICU, we performed propensity score matching to minimize the confounding effects due to non-randomized assignment. The factors selected for propensity score matching covered indicators previously reported in the literature [3, 22, 23] or clinically considered to be important to the outcome, including demographic data, smoking history, drinking history, ASA classification, comorbidities, etiology of end-stage liver diseases, MELD score, preoperative artificial liver support, basic SpO_2_, preoperative laboratory data and intraoperative findings. Based on these factors, the baseline variables were well balanced between the two groups after matching. In matched analyses, the results showed that the composite mechanical ventilation-related adverse outcomes occurred less frequently in the OR extubation group. Moreover, the above results were verified with logistic regression analysis. Not surprisingly, the result of logistic regression analysis was in line with matched analysis (see Additional file 7: Fig. S2). However, when analyzing each individual event that made up the composite mechanical ventilation-related adverse outcomes, no significant differences were found between the two groups, which may be due to the small sample size and not having enough power to detect clinically meaningful increases in the risk of adverse outcomes. Additionally, given the lack of available evidence, this finding is considered exploratory at this point, and future larger studies are necessary to further elucidate this aspect.

Shortening the length of ICU stay is also crucial for improving patients' prognosis since long-term ICU stay may lead to long-lasting physical, cognitive and/or mental health impairments. In this study, we found an association between extubation in the OR and shorter ICU length of stay, as well as less total hospital cost, which was consistent with previous studies [16, 24, 25]. At our center, patients undergoing liver transplantation had a relatively fixed cost of preoperative imaging and laboratory examination, drugs, surgery and anesthesia. The cost that made a difference was primarily from postoperative treatment, especially that on mechanical ventilation in the ICU and surgical complications after surgery. This was further reinforced by the finding that the cost was increased when patients were extubated in the ICU ((median 3.9, IQR (3.5 ~ 4.6) 10000 US dollars *vs.* median 4.1, IQR (3.8 ~ 5.1) 10000 US dollars). Therefore, early extubation contributed to a shorter length of ICU stay after surgery and subsequently less cost. In line with these findings, a retrospective study focusing on patients undergoing liver transplantation with FastTrack protocol involving extubation in the OR also resulted in a relatively shorter ICU stay and cost without increasing pulmonary complications and reintubation rates [16]. Another study that included 64 pediatric liver transplant patients also demonstrated that OR extubation led to shorter ICU stay and earlier hospital discharge [24]. In a more broad sense, early discharge from the ICU also increases the number of beds open to other patients in need of intensive care.

In order to exclude the major differences in patient baseline characteristics, which might be responsible for the late extubation and subsequent worse postoperative outcomes and prolonged ICU length of stay and total hospital cost, we made a propensity score-matched analysis. However, there was still a small portion of extubation in the ICU mechanical time more than 24h after matching. Thereafter, a comparison between patients who were extubated in the OR and in the ICU within 24h was performed after excluding the patients extubated later than 24h postoperatively. The results still showed a significant decrease in composite mechanical ventilation-related adverse outcomes, ICU length of stay, and total hospital cost (see Additional file 8: Table S6).

Extubation in the OR has been proved to be safe in most liver transplantation recipients [15]. Consistent with previous studies [26], our results showed that the unplanned reintubation rates in the OR extubation group were not increased compared to patients extubated in ICU. Moreover, in most reintubation cases, the causes of early reintubation were related to urgent reoperations, whereas inadequate reawakening or respiratory failures were rarely implicated.

Logistic regression analysis of our data revealed that patient’s smoking history, duration of surgery, intraoperative PRBC transfusion, and the maximal dose of vasopressors were significantly associated with OR extubation. One of the novel findings in our study is that patients without smoking history showed a significantly higher likelihood of early extubation. This may be due to the fact that patients without smoking history might have better lung function, which in turn might lead to a higher likelihood of early extubation in this population. With regard to the duration of surgery, as described by Mandell *et al* [27], a longer duration of surgical procedure was associated with fewer attempts at early extubation. Additionally, many studies have focused on intraoperative hemodynamic instability for predicting early extubation after liver transplantation [26], and our results also showed that intraoperative factors related to hemodynamic disturbance (*i.e*., the use of vasopressor, and a requirement for a large PRBC transfusion) were negatively associated with early extubation. A retrospective study by Skurzak et al. [26] suggested that patients intraoperative PRBC transfusion <7 units and norepinephrine <0.05 μg/kg/min at the end of surgery were more suitable for OR extubation. MELD score is considered a valuable tool for identifying the sickest patients. However, it remains controversial whether the MELD score can be utilized as one of the extubation criteria. Similarly to some previous studies, we found that MELD score had no impact on early extubation [28, 29]. On the contrary, a prospective study by Biancofiore et al. [30] suggested that a MELD score of 11 points was the optimal cut-off for immediate postoperative extubation, but the sensibility and specificity were found to be low. Thus, even patients with higher MELD score may not necessarily need to be excluded from an early extubation protocol before the beginning of surgery. Based on our results and previous studies, it was shown that patients without a smoking history, short surgery time, and/or intraoperative hemodynamic stability showed a higher likelihood of operating room extubation. Therefore, for patients with above characteristics, we recommend to extubate them in the OR.

This study has several limitations. Firstly, this study was a non-randomized, observational rather than experimental study. We, therefore, performed propensity score matching to reduce potential bias and strengthen our reported effect estimates. However, it is inevitable that the use of propensity score matching will shrinking the sample size. Secondly, the lack of some data (*e.g*., cold ischemia time, as well as donor factors such as donor steatosis, Donation after circulatory death/Donation after Brain Death) could not be avoided because of the study's retrospective design. However, the most relevant factors that might impact the outcomes were involved in the current study. Thirdly, the judgments about tracheal extubation were subjective to some degree [25]. Thus, a standardized protocol is essential for early extubation. Fourthly, we acknowledge that the numbers of patients evaluated in this study were small. Moreover, to preserve the stability of the multivariable model, we had to limit the number of variables in the final model. We selected 22 variables for the initial univariable model. We assessed correlation among those 22 factors and eventually restricted the maximum number to 9 variables that were included in the first multivariable model. Finally, because this analysis comprised a 3-year time span of liver transplantation, some changes in perioperative procedures occurred during this time period. Therefore, further large prospective matched studies are required to determine whether early extubation has positive effects on adverse clinical outcomes.

## Conclusions

Our data suggested that the practice of early extubation in the OR is associated with a lower incidence of composite mechanical ventilation-related adverse outcomes, shorter ICU stay, and lower total hospital cost without increasing the reintubation rates. Given the benefits of early extubation in the OR, this practice should be considered when appropriate. Meanwhile, it is important to identify liver transplantation recipients eligible for early extubation in the OR.

## Supplementary Information

**Additional file 1: Table S1.** STROBE Statement. Checklist of our cohort study, which demonstrates STROBE Statement Checklist of our cohort study.

**Additional file 2: Table S2** Predefined Acute Kidney Injury (AKI) According Kidney Disease Improving Global Outcomes Guidelines (KIDGO).

**Additional file 3: Table S3** Predefined Postoperative Complications According European Perioperative Clinical Outcome definitions.

**Additional file 4: Figure S1** Histograms of the estimated propensity scores.

**Additional file 5: Table S4** Postoperative complications after matching.

**Additional file 6: Table S5** Unplanned-Reintubation indications amongst OR extubation and ICU extubation groups after matching.

**Additional file 7: Figure S2** Association between extubation in the OR and composite mechanical ventilation-related adverse outcomes.

**Additional file 8: Table S6** Outcomes between OR extubation group and ICU extubation group (within 24h) after matching.

## Data Availability

The datasets used and/or analyzed during this study are available from the corresponding author on reasonable request.

## Abbreviations

OR: Operating Room
ICU: intensive care unit
AKI: Acute Kidney Injury
BMI: Body Mass Index
ASA: American Society of Anesthesiologists
MELD: Model for End-Stage Liver Disease
INR: International Normalized Ratio
WBC: White Blood Cell
PT: Prothrombin Time
APTT: Activated Partial Thromboplastin Time
PRBC: Packed Red Blood Cells
