# Supplementary material for: Extubation in the operating room results in fewer composite mechanical ventilation-related adverse outcomes in patients after liver transplantation: a retrospective cohort study
Source: BMC Anesthesiol. 2021 Nov 18;21:286. doi: 10.1186/s12871-021-01508-1 (PMC8600887; doi:10.1186/s12871-021-01508-1)
Supplement: Supplementary file 2 — Additional file 2: Table S2 Predefined Acute Kidney Injury (AKI) According Kidney Disease Improving Global Outcomes Guidelines (KIDGO). [file 12871_2021_1508_MOESM2_ESM.docx]

**Table S2. Predefined Acute Kidney Injury (AKI) According Kidney Disease Improving Global Outcomes Guidelines(KIDGO)**

| **Stage** | **Serum creatinine** | **Urine output** |
| --- | --- | --- |
| 1 | 1.5-1.9 times baseline value within 7days or ≥27μmol/L(0.3mg/dl) increase within 48h | <0.5ml/kg/h for 6-12h |
| 2 | 2.0-2.9 times baseline value within7 days | <0.5ml/kg/h for 12h |
| 3 | 3.0 times baseline within 7days or increase in serum creatinine to ≥354μmol/L(≥4.0mg/dl with an acute rise of >44μmol/L(0.5mg/dl) or initiation of renal replacement therapy or in patients <18 years, decrease in eGFR to <35ml/min per1.73m^2^ | <0.3ml/kg/h for 24h or Anuria for 12h |
