# Supplementary material for: Extubation in the operating room results in fewer composite mechanical ventilation-related adverse outcomes in patients after liver transplantation: a retrospective cohort study
Source: BMC Anesthesiol. 2021 Nov 18;21:286. doi: 10.1186/s12871-021-01508-1 (PMC8600887; doi:10.1186/s12871-021-01508-1)
Supplement: Supplementary file 3 — Additional file 3: Table S3 Predefined Postoperative Complications According European Perioperative Clinical Outcome definitions. [file 12871_2021_1508_MOESM3_ESM.docx]

**Table S3. Predefined Postoperative Complications According European Perioperative Clinical Outcome definitions**

| **Complication** | **Definition** | **Graduation** |
| --- | --- | --- |
| **Respiratory failure** | Presence of the following manifestations (PaO2<60 mmHg on room air, ratio of PaO2 to inspired oxygen fraction <300, or oxygen saturation <90%) and required therapeutic intervention (oxygen therapy or mechanical ventilation) for more than 24 hours. | ***Mild:*** Results in only temporary harm and would not usually require specific clinical treatment.  ***Moderate:*** More serious complication but one which does not usually result in permanent harm or functional limitation. Usually requires clinical treatment.  ***Severe:*** Results in significant prolongation of hospital stay and/or permanent functional limitation or death. Almost always requires clinical treatment. |
| **Pleural effusion** | Confirmed by chest X-ray or ultrasound examination and required therapeutic intervention (drainage, aspiration, and/or diuresis after albumin administration). | ***Mild:*** Results in only temporary harm and would not usually require specific clinical treatment.  ***Moderate:*** More serious complication but one which does not usually result in permanent harm or functional limitation. Usually requires clinical treatment.  ***Severe***: Results in significant prolongation of hospital stay and/or permanent functional limitation or death. Almost always requires clinical treatment. |
| **Pulmonary infection** | Presence of at least one of the following manifestations (increased or color-changed sputum, new or changed pulmonary infiltrates, fever, leukocyte count > 12,000/mm3) and required antibiotic therapy. | ***Mild***: Results in only temporary harm and would not usually require specific clinical treatment.  ***Moderate***: More serious complication but one which does not usually result in permanent harm or functional limitation. Usually requires clinical treatment.  ***Severe***: Results in significant prolongation of hospital stay and/or permanent functional limitation or death. Almost always requires clinical treatment. |
| **Surgical site infection (superficial)** | Infection involving only superficial surgical incision which meets the following criteria:  1)Infection occurs within 30 days after surgery and  2)Involves only skin and sub-cutaneous tissues of the incision and  3)The patient has at least one of the following:  a) Purulent drainage from the superficial incision  b) Organisms isolated from an aseptically obtained culture of fluid or tissue from the superficial incision and at least one of the following signs or symptoms of infection: pain or tenderness, localized swelling, redness, or heat, or superficial incision is deliberately opened by surgeon and is culture positive or not cultured. A culture- negative finding does not meet this criterion.  c) Diagnosis of a incisional surgical site infection by a surgeon or attending physician | ***Mild:*** Results in only temporary harm and would not usually require specific clinical treatment.  ***Moderate:*** More serious complication but one which does not usually result in permanent harm or functional limitation. Usually requires clinical treatment.  ***Severe:*** Results in significant prolongation of hospital stay and/or permanent functional limitation or death. Almost always requires clinical treatment. |
| **Surgical site infection (deep)** | An infection which involves both superficial and deep parts of surgical incision and meets the following criteria:  1)Infection occurs within 30 days after surgery if no surgical implant is left in place or one year if an implant is in place and  2)The infection appears to be related to the surgical procedure and involves deep soft tissues of the incision (e.g. fascial and muscle layers) and  3)The patient has at least one of the following: a) Purulent drainage from the deep incision but not from the organ/space component of the surgical site  b) A deep incision spontaneously dehisces or is deliberately opened by a surgeon and is culture-positive or no cultures were taken whilst the patient has at least one of the following signs or symptoms of infection: fever (>38°C) or localized pain or tenderness. A culture-negative finding does not meet this criterion.  c) An abscess or other evidence of infection involving the deep incision is found on direct examination, during surgery, or by histopathologic or radiologic examination d) Diagnosis of a deep incisional surgical site infection by a surgeon or attending physician | ***Mild:*** Results in only temporary harm and would not usually require specific clinical treatment.  ***Moderate***: More serious complication but one which does not usually result in permanent harm or functional limitation. Usually requires clinical treatment.  ***Severe:*** Results in significant prolongation of hospital stay and/or permanent functional limitation or death. Almost always requires clinical treatment. |
| **Surgical site infection (organ/space)** | An infection which involves any part of the body excluding the fascia or muscle layers and meets the following criteria:  1) Infection occurs within 30 days after surgery and  2) The infection appears to be related to the surgical procedure and involves any part of the body, excluding the skin incision, fascia, or muscle layers, that is opened or manipulated during the operative procedure and  3) The patient has at least one of the following:  a) Purulent drainage from a drain that is placed through a stab wound into the organ/space  b) Organisms isolated from an aseptically obtained culture of fluid or tissue in the organ/ space  c) An abscess or other evidence of infection involving the organ/space that is found on direct examination, during reoperation, or by histopathologic or radiologic examination  d) Diagnosis of an organ/space surgical site infection by a surgeon or attending physician | ***Mild***: Results in only temporary harm and would not usually require specific clinical treatment.  ***Moderate***: More serious complication but one which does not usually result in permanent harm or functional limitation. Usually requires clinical treatment.  ***Severe:*** Results in significant prolongation of hospital stay and/or permanent functional limitation or death. Almost always requires clinical treatment***.*** |
| **Bloodstream infection** | An infection which is not related to infection at another site and which meets either of the following criteria:  1)Patient has a recognised pathogen cultured from blood cultures which is not related to an infection at another site  2)Patient has at least one of the following signs or symptoms: fever (>38°C), chills, or hypotension and at least one of the following:  a) Common skin contaminant cultured from two or more blood cultures drawn on separate occasions  b) Common skin contaminant cultured from at least one blood culture from a patient with an intravascular line, and a physician starts antimicrobial therapy  c) Positive blood antigen test | ***Mild***: Results in only temporary harm and would not usually require specific clinical treatment.  ***Moderate***: More serious complication but one which does not usually result in permanent harm or functional limitation. Usually requires clinical treatment.  ***Severe:*** Results in significant prolongation of hospital stay and/or permanent functional limitation or death. Almost always requires clinical treatment. |
