# Supplementary figures and images for: Extubation in the operating room results in fewer composite mechanical ventilation-related adverse outcomes in patients after liver transplantation: a retrospective cohort study
Source: BMC Anesthesiol. 2021 Nov 18;21:286. doi: 10.1186/s12871-021-01508-1 (PMC8600887; doi:10.1186/s12871-021-01508-1)

**Figure S1. Histograms of the estimated propensity scores**


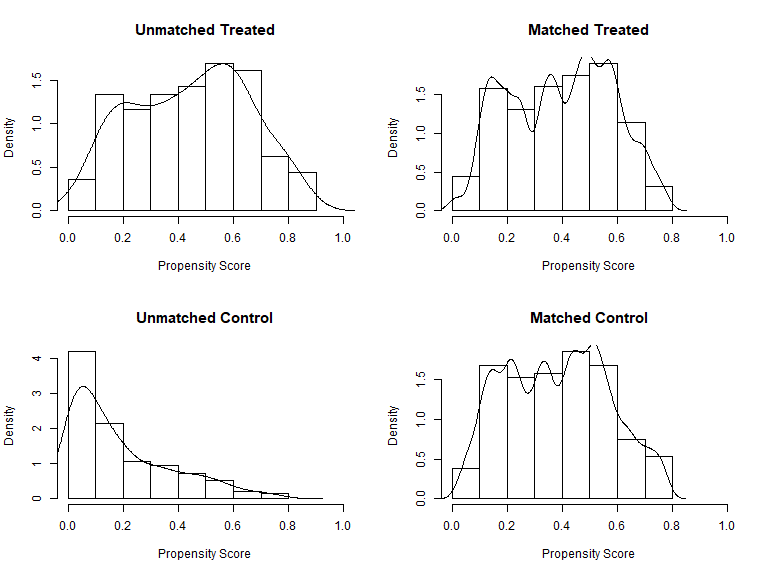

Supplement: Supplementary file 4 — Additional file 4: Figure S1 Histograms of the estimated propensity scores. [file 12871_2021_1508_MOESM4_ESM.docx]
