# Supplementary material for: Extubation in the operating room results in fewer composite mechanical ventilation-related adverse outcomes in patients after liver transplantation: a retrospective cohort study
Source: BMC Anesthesiol. 2021 Nov 18;21:286. doi: 10.1186/s12871-021-01508-1 (PMC8600887; doi:10.1186/s12871-021-01508-1)
Supplement: Supplementary file 5 — Additional file 5: Table S4 Postoperative complications after matching. [file 12871_2021_1508_MOESM5_ESM.docx]

**Table S4. Postoperative complications after matching**

| **Outcome** | **Propensity-matched Cohort** | | | |
| --- | --- | --- | --- | --- |
|  | **OR Extubation** | **ICU Extubation** | **Odds Ratio (95%CI)** | ***P* Value** |
|  | **n=94** | **n=148** |  |  |
| **30-day all causes mortality, AKI (**Stage 2 or 3**), Moderate-severe pulmonary complications** |  |  |  |  |
| 30-day all causes mortality | 4(4.3%) | 16(10.8%) | 0.367(0.119-1.133) | 0.081 |
| AKI (stage 2 or 3) | 0 | 5(3.4%) | / | 0.181 |
| Moderate to severe pulmonary complications | 17(18.1%) | 35(23.6%) | 0.713(0.373-1.362) | 0.306 |
| Respiratory failure | 0 | 3(2.0%) | / | 0.428 |
| Pulmonary infection | 9(9.6%) | 19(12.8%) | 0.719(0.311-1.664) | 0.441 |
| Pleural effusion | 8(8.5%) | 24(16.2%) | 0.481(0.206-1.120) | 0.090 |
| **Moderate to severe Infectious complications** |  |  |  |  |
| Surgical site infection (superficial/deep, organ/ space) | 0 | 4(2.7%) | / | 0.276 |
| Pulmonary infection | 9(9.6%) | 19(12.8%) | 0.719(0.311-1.664) | 0.441 |
| bloodstream infection | 2(2.1%) | 3(2.0%) | 1.051(0.172-6.409) | 0.957 |

Data are presented as mean ± SD, median (IQR), or number of patients (percentage) and compared by independent samples t-test, Mann-Whitney U test or chi-squared test/ Fisher’s exact test respectively.

OR,operating room; ICU, intensive care unite; CI, confidence index; AKI, acute kidney injury.
