# Supplementary material for: Extubation in the operating room results in fewer composite mechanical ventilation-related adverse outcomes in patients after liver transplantation: a retrospective cohort study
Source: BMC Anesthesiol. 2021 Nov 18;21:286. doi: 10.1186/s12871-021-01508-1 (PMC8600887; doi:10.1186/s12871-021-01508-1)
Supplement: Supplementary file 6 — Additional file 6: Table S5 Unplanned-Reintubation indications amongst OR extubation and ICU extubation groups after matching. [file 12871_2021_1508_MOESM6_ESM.docx]

**Table S5 Unplanned-Reintubation indications amongst OR extubation and ICU extubation groups after matching**

| **Unplanned-reintubation indication** | **OR extubation group(n=6)** | **ICU extubation group(n=20)** |
| --- | --- | --- |
| Respiratory Distress/ Hypoxia | 1 | 5 |
| Altered Mental status | / | 1 |
| Unplanned-Reoperation | 4 | 11 |
| Cardiovascular instability | 1 | 3 |

OR,operating room; ICU, intensive care unite
