# Supplementary material for: Extubation in the operating room results in fewer composite mechanical ventilation-related adverse outcomes in patients after liver transplantation: a retrospective cohort study
Source: BMC Anesthesiol. 2021 Nov 18;21:286. doi: 10.1186/s12871-021-01508-1 (PMC8600887; doi:10.1186/s12871-021-01508-1)
Supplement: Supplementary file 7 — Additional file 7: Figure S2 Association between extubation in the OR and composite mechanical ventilation-related adverse outcomes. [file 12871_2021_1508_MOESM7_ESM.docx]

**Figure S2. Association between extubation in the OR and composite mechanical ventilation-related adverse outcomes^†^**

**
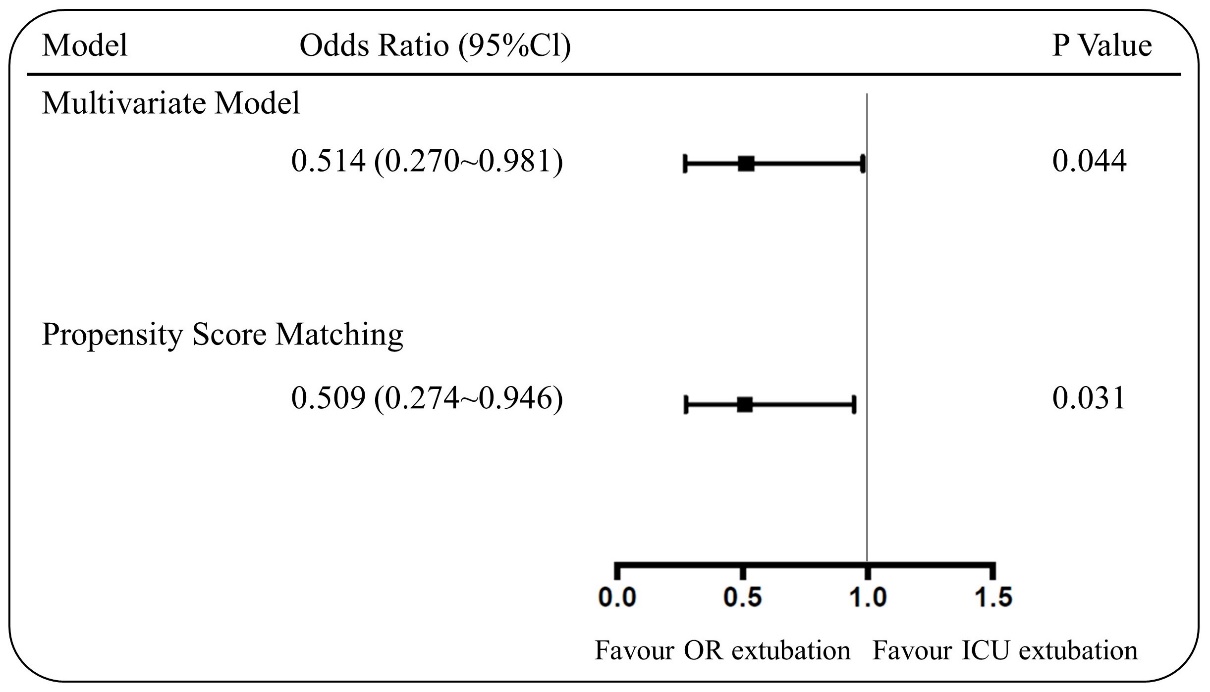
**

OR, operating room; ICU, intensive care unite; CI, Confidence index.

^†^Composite mechanical ventilation-related adverse outcomes includes 30-day all-cause mortality, in-hospital acute kidney injury (stage 2 or 3), or in-hospital moderate to severe pulmonary complications.
