# Supplementary material for: Extubation in the operating room results in fewer composite mechanical ventilation-related adverse outcomes in patients after liver transplantation: a retrospective cohort study
Source: BMC Anesthesiol. 2021 Nov 18;21:286. doi: 10.1186/s12871-021-01508-1 (PMC8600887; doi:10.1186/s12871-021-01508-1)
Supplement: Supplementary file 8 — Additional file 8: Table S6 Outcomes between OR extubation group and ICU extubation group (within 24h) after matching. [file 12871_2021_1508_MOESM8_ESM.docx]

**Table S6. Outcomes Between OR extubation group and ICU extubation group (within 24h) after matching**

| **Outcome** | **Propensity-matched Cohort** | | **Odds Ratio 95% CI** | **P** |
| --- | --- | --- | --- | --- |
|  | **OR extubation group**  **(n=94)** | **ICU extubation group (within 24h)**  **(n=133)** |  |  |
| **Primary Outcome** |  |  |  |  |
| 30-day all cause mortality, AKI (stage 2 or 3), Moderate to severe pulmonary complications | 18(19.1%) | 42(31.6%) | 0.513(0.273~0.964) | 0.036 |
|  |  |  |  |  |
| **Secondary Outcome** |  |  |  |  |
| Moderate to severe infectious complications | 9(9.6%) | 22(16.5%） | 0.534(0.234~1.219) | 0.132 |
| ICU length of stay | 4(3~6) | 5(4~8) | / | <0.001 |
| Hospital length of stay | 12(10~17) | 13(10~19) | / | 0.210 |
| Unplanned-reintubation | 6(6.4%) | 19(14.3%) | 0.409(0.157~1.067) | 0.061 |
| Total hospital cost | 3.8(3.5~4.6) | 4.2(3.8~5.1) | / | 0.031 |

Data are presented as mean ± SD, median (IQR), or number of patients (percentage) and compared by independent samples t-test, Mann-Whitney U test or chi-squared test/ Fisher’s exact test respectively.

OR, operating room; ICU, intensive care unite; CI, Confidence index; AKI, acute kidney injury.
